# Management of Non-A Non-B Aortic Dissection: A Narrative Review

**DOI:** 10.3390/jcdd12010001

**Published:** 2024-12-24

**Authors:** Joseph Kletzer, Stoyan Kondov, Aleksandar Dimov, Victoria Werdecker, Martin Czerny, Maximilian Kreibich, Tim Berger

**Affiliations:** 1 Department of Cardiovascular Surgery, University Hospital Freiburg Heart Centre, 79106 Freiburg, Germany; stoyan.kondov@uniklinik-freiburg.de (S.K.); aleksandar.dimov@uniklinik-freiburg.de (A.D.); victoria.werdecker@uniklinik-freiburg.de (V.W.); martin.czerny@uniklinik-freiburg.de (M.C.); maximilian.kreibich@uniklinik-freiburg.de (M.K.); tim.berger@uniklinik-freiburg.de (T.B.); 2 Faculty of Medicine, University of Freiburg, 79085 Freiburg, Germany

**Keywords:** aortic dissection, non-a non-b dissection, tevar, open-surgery

## Abstract

Non-A non-B aortic dissection remains a complex and controversial topic in cardiovascular management, eliciting varied approaches among cardiologists and surgeons. Due to the limited evidence surrounding this condition, existing guidelines are limited in the complexity of their recommendations. While most patients are initially managed medically, invasive treatment becomes necessary in a large proportion of patients. When surgery is considered, the most utilized techniques include the frozen elephant trunk procedure and endovascular repair strategies targeting the arch and descending thoracic aorta. This narrative review aims to synthesize current knowledge and clinical experiences, highlighting the challenges and evolving practices related to non-A non-B dissection management.

## 1. Introduction

Since it was first described by Pasic et al. in 1999, non-A non-B aortic dissection has been an elusive issue in the realm of aortic disease [1]. While historically commonly used classifications systems such as the DeBakey and Stanford classifications have concentrated mostly on the ascending and descending aorta, one crucial section was mostly ignored. The aortic arch, while rarely being the origin of a dissection compared to other sections of the thoracic aorta, is a key segment, with the potential for threatening complications once involved in a dissection. Only recently, with the acceptance of a contemporary classification system considering type of dissection, entry site, and malperfusion (TEM), has the aortic arch found recognition in the definition of the dissection type [2,3,4]. Available data suggests that these non-A non-B dissections make up approximately 2–11% of aortic dissections [5,6]. Due to an apparent high rate of severe complications such as stroke or organ malperfusion if left untreated, early surgery has been suggested to be necessary in around 19% of non-A non-B dissections [2]. That said, more than 50% of patients with this pattern of dissection are initially managed medically.

The issue regarding conservative versus surgical management of non-A non-B dissections is a highly relevant and timely matter. Being at the border between type B dissections, which in the absence of certain risk factors have historically been treated with best medical therapy, and type A dissections, which are always an emergent indication for surgery, the appropriate treatment of non-A non-B dissections remains controversial [2]. While some retrospective analyses contribute improved outcomes to early surgical management, large prospective trials remain to be conducted [7]. Moreover, although current European association of cardiothoracic surgery/Society of thoracic surgeons (EACTS/STS) guidelines attempt to give substantiated guidance for the management of this rare subtype of dissection, available evidence is sparse, leading to mostly expert opinion-based recommendations [2]. Most likely owing to this limitation, in the recent European society of cardiology (ESC) guidelines, advice on the treatment of non-A non-B dissection was mostly sidelined to a single subparagraph below the recommendations regarding management of malperfusion [3].

Consequently, this review aims to expand upon the management of non-A non-B dissection and give an overview of current treatment modalities.

## 2. Indication for Treatment

When managing patients with a new diagnosis or progress of non-A non-B dissection, weighing patient-specific risk posed by surgery or conservative treatment is substantial for generating optimal outcomes. Due to its morphological proximity to type B dissections, uncomplicated non-A non-B dissections have historically been treated conservatively [6]. Current guidelines reflect this notion. However, once the patient exhibits signs of dissection-associated complications or high-risk factors thereof, emergent, or at least urgent, surgical treatment is indicated [2]. Criteria for complicated non-A non-B dissection were defined as follows:

1. Dissection-associated malperfusion
2. Pleural effusion containing blood suggesting rupture
3. Contained and/or free aortic rupture
4. Persistent pain
5. Uncontrollable arterial hypertension

Emergent treatment of these types of dissection is always indicated due to the immediate risk of death, organ failure, or rapid disease progression [2]. Of all in-hospital deaths, it is estimated that 7% of patients suffered from visceral ischemia, 18% from aortic rupture, and 29% from neurological dysfunction [5].

On the other hand, in uncomplicated dissection, patients with probable disease progression must be filtered out and treated in a non-emergent setting to improve outcomes. Several risk factors have been formed especially for type B dissection but were also used in recommendations for non-A non-B dissections. Aside from a primary entry size > 10 mm, proximity of the primary entry to the left subclavian artery, false lumen diameter > 22 mm, descending aortic diameter > 40 mm, or high antegrade flow volume with significant retrograde flow assessed in magnetic resonance tomography, location of the primary entry tear at the inner curvature of the aortic arch has been included as a morphological high-risk feature [2,8]. As opposed to the other features, it has been postulated to be an independent risk factor for retrograde Type A dissection, due to the lacking physical barrier created by the supra-aortic vessels at the outer curvature [8]. As these high-risk features are associated with a high probability of disease progression, but no tissue damage per se, surgery is indicated within 48 h, as opposed to immediately in complicated dissections. The treatment modality is then decided on after taking into consideration dissection morphology and patient operability, as well as general vascular status [2].

## 3. Open Treatment

Once invasive treatment for non-A non-B dissection is indicated, therapeutic principles mostly stay the same. As in every other type of dissection, a tear-oriented approach should be applied to exclude especially the primary entry tear from circulation. This has been shown to increase favorable remodeling in other types of dissection [9,10]. As the location of the primary entry tear is depicted in the TEM classification, current recommendations for surgical treatment are classified according to this. Firstly, should a primary entry tear not be detectable, meaning the dissection is classified as non-A non-B E-0, optimal medical treatment with tight blood pressure control and computed tomography angiography (CTA) imaging during follow-up for the early detection of any disease progression is indicated. As evidence on the optimal follow-up interval in such cases is nigh non-existent, they should be scheduled as per clinical judgement and patient preferences. Once the location of the primary entry tear has been pinpointed, surgical treatment is often indicated. In case of entry in the arch, meaning a non-A non-B E-2 situation, surgical treatment via the frozen elephant trunk (FET) technique is indicated.

### 3.1. Frozen Elephant Trunk (FET)

**Figure 1:**
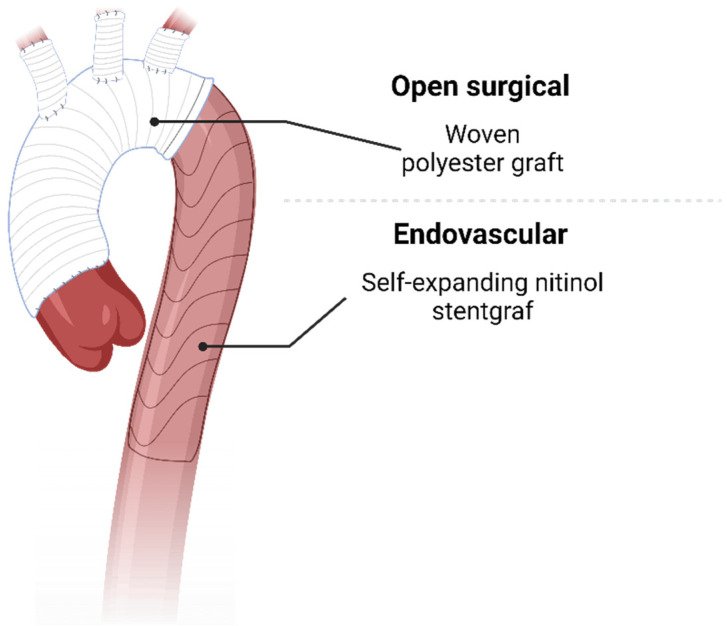
Visualization of FET implants showing the open surgically implanted woven prosthesis part, as well as the endovascular stent graft.

The FET technique is an open surgical technique where a hybrid aortic prosthesis with a stent graft component connected to an aortic arch prosthesis which is introduced to the diseased aorta under direct vision or guided by a wire is implanted (Figure 1). This technique, given the indication, may be extended to include aortic root replacement without significantly increased perioperative risk, if performed in a high-volume aortic center [11,12]. Additionally, FET can also be performed with beating heart technique [13]. As cerebral perfusion is limited during the procedure, intraoperative selective antegrade cerebral perfusion (SACP) is recommended. To ensure adequate perfusion strategy, preoperative imaging of the cerebral vasculature, specifically the circle of willis, should be acquired if possible, as failure to do so might result in a worse neurological outcome. Epidemiologic data have shown that in about 4.5% of all aortic dissection, arch anomalies are present which may cause hypoperfusion in unilateral selective antegrade cerebral perfusion (SACP) [14]. However, these data also suggest that unilateral SACP potentially leads to adequate cerebral perfusion in over 90% of patients with aortic dissection. Modifications include bilateral or even trilateral SACP for adequate cerebral protection based on preoperative imaging [15,16]. Still, in many cases, preventive bilateral SACP may be warranted based on the complexity of the case, to account for unforeseen circumstances. However, as it is estimated that 73.8% of strokes during FET are of embolic origin, adequate protection against cerebral malperfusion cannot completely reduce the risk of stroke for these patients. Therefore, minimal manipulation, as well as cautious deairing, should always be practiced in addition to SACP [17]. For even greater safeguarding against ischemia, surgery in moderate hypothermic arrest as well as lower-body perfusion via an extracorporal circulation branch of the implanted prosthesis may be used [18]. For adequate sizing, an increase in the aortic diameter of 20% should be assumed. In the descending thoracic aorta, Rylski et al. have shown that the diameter increases by about 3.7 mm, when comparing CTA before dissection to right after [19]. Adequate oversizing seems paramount to avoid type Ib endoleak. Still, in case of doubt, to avoid distal-stent-graft-induced new entry (dSINE), undersizing of the endovascular portion of the implanted prosthesis may be used [20,21].

Outcomes after utilizing FET for the treatment of non-A non-B dissection are estimated to be around 5% for operative mortality and to increase to about 7.1% during the first 30 days after surgery [22,23]. One-year mortality is suggested to be around 18% by Luo et al. [22]. During follow-up, subsequent distal reintervention might be needed in about 20.7% of patients over 7 years [22]. This may be due to reasons such as type Ib endoleak caused by dSINE or similar etiologies such as changes in the geometry of more distal aortic segments [24]. Robust risk factors for the prediction of these reinterventions, however, have not been identified [25]. Especially dSINE is an important complication to consider, as it may develop at any time after the implantation of the FET, and there are again no parameters accurately predicting the occurrence of dSINE, especially after aortic dissection [26,27]. In the case of multisegmented dissecting aneurysm in non-A non-B dissection, a planned multistage approach may be utilized, which has been associated with favorable clinical outcomes [28,29]. However, this has not been sufficiently investigated in the context of non-A non-B aortic dissection. Lastly, another rare presentation of complication after FET is new thrombus formation within the FET stent graft. Such formations occur in around 6% of FET procedures, mostly in the distal segment of the prosthesis. Should they embolize, it may even lead to the demise of the patient through mechanisms such as visceral ischemia [30].

More novel approaches to the FET technique include new types of prostheses such as an FET with a trifurcated side branch, which enables proximalization of the distal anastomosis, which possibly converts the FET into a more minimally invasive technique [31]. Another way to proximalize the anastomosis is an endovascular side branch attached to the FET prosthesis, which is supposed to be inserted into the left subclavian artery and which would also potentially reduce the time of operation, as one fewer anastomosis would need to be completed [32].

### 3.2. Conventional Total Arch Replacement

As the FET technique has become available, in most cases conventional total arch replacement has become all but obsolete in the management of non-A non-B dissection. Most importantly, current guidelines have even left out the possibility of this non-hybrid approach in their recommendations [2,3,33]. In a recent meta-analysis by Carino et al., it is estimated that standard arch replacement is performed in merely 3% of non-A non-B dissection with a high degree of between-center variance [34]. This might be feasible in very limited instances of dissection, limited only to the aortic arch. If performed in a dissection extending beyond the arch, thoracic endovascular aortic repair (TEVAR) has been shown to be necessary in most cases [5,35]. Therefore, when performing a conventional aortic arch replacement, the creation of a sufficient landing zone is an I-C recommendation in current guidelines [2]. However, with the availability of FET, planned TEVAR during follow-up after conventional arch replacement seems rather unnecessary and would need a very specific indication.

## 4. Endovascular Treatment

Abiding by a tear-oriented approach as described previously, should the primary entry tear be located somewhere in the descending aorta, making the dissection E-3, TEVAR might be suitable if anatomically feasible. This is mostly dependent on an available landing zone. Current recommendations specify at least 20 mm from the beginning of the landing zone to the entry location [2]. These landing zones may also be artificially extended using various techniques described in the subsequent sections. Currently, TEVAR is the most common treatment modality used for invasive treatment of non-A non-B dissection [34]. The indication might even be extended, as some recent studies have suggested TEVAR to be feasible in patients with hereditary thoracic aortic disease, which due to the questionable stability of the landing zone was previously thought to be impractical [36,37].

### 4.1. Carotid Subclavian Bypass/Extrathoracic Transposition

When extending the native landing zone in non-A non-B dissection, extrathoracic transposition of the supra-aortic vessels, mostly carotid subclavian bypass, is the most commonly used method [34]. It is indicated in cases where a transposition of the vessels, and therefore exclusion of the most proximal section of the left subclavian artery from the remaining circulation, would lead to a potential landing zone > 25 mm for TEVAR. Additional indications may include the impossibility of aortic cross clamping due to soft-plaque or extensive aortic calcification at the proximal aorta. It also may be used to avoid cardiopulmonary bypass and hypothermic circulatory arrest in patients with high intraoperative risk [2]. Carotid subclavian bypass can be performed using different techniques. Firstly, the left subclavian artery may be detached from its origin at the aortic arch and directly attached to the left common carotid artery. This technique is commonly directly referred to a left subclavian artery transposition. Another practicable method to achieve exclusion of the proximal left subclavian artery is to use polytetrafluoroethylene (PTFE) or dacron grafts as an extraanatomical bypass, to link together the common carotid and left subclavian artery. Reversed saphenous veins may also be used using the same method [38]. Owing to its partly open surgical nature, TEVAR together with carotid subclavian bypass surgery carries a specific complication risk profile as opposed to TEVAR alone. Due to anatomical proximity, several nerve palsies have been described. Phrenic nerve palsy was described in 25%, recurrent laryngeal nerve palsy in 5%, and axillary nerve palsy in 2% of patients. However, permanent paresis was only reported in 0.9% of patients. More severe complications include neck hematoma requiring re-exploration, which was reported in 5% of cases. Generally speaking, carotid subclavian bypass surgery had favorable outcomes with a high technical success rate and a reported 5-year graft patency of about 97% [39,40].

### 4.2. Total Endovascular Approach

**Figure 2:**
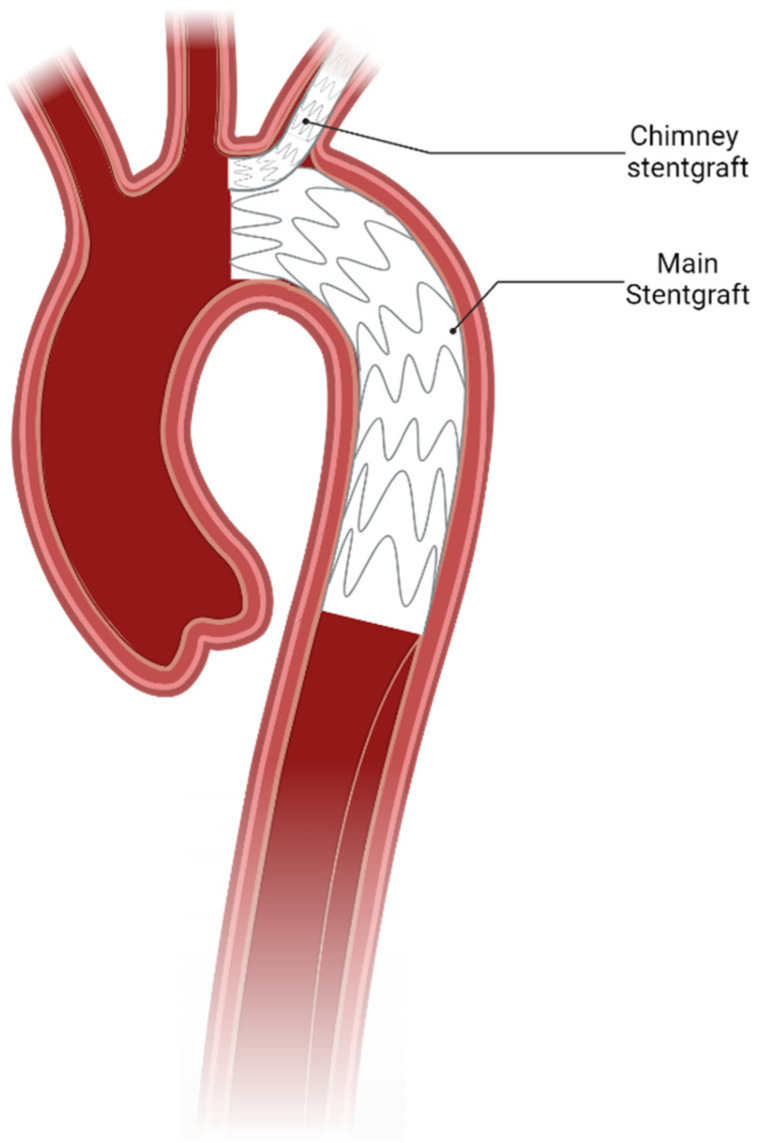
Illustration of the chimney graft technique showing a smaller endovascular stent graft implanted tangential to the main aortic stent graft to supply the left subclavian artery with blood flow.

Another possibility to enhance endovascular indication in patients with limited conventional landing zones is to use endovascular branching, fenestration, or so-called chimney grafts to avoid covering supra-aortic vessels (Figure 2). However, due to insufficient experience, in current guidelines, these endovascular treatment modalities do not appear in conjunction with non-A non-B dissection [2,3].

Firstly, to avoid the need for carotid subclavian bypass surgery, a separate off-the-shelf stent graft may be implanted in parallel to the main aortic stent graft connecting the left subclavian artery to aortic circulation while still allowing the main aortic landing zone to be placed more proximally to the left subclavian artery. One major concern after using this type of aortic repair is the so-called gutter endoleak, which is a type of Ia endoleak resulting from a displacement of the main aortic stent graft by the chimney stent graft at the proximal landing zone leading to an incomplete adherence. While the technical success rate was high, the early-type Ia endoleak rate of this method ranges from 6.5% to 13%, with a 30-day mortality of 7.9%, a reintervention rate of 10.6%, and a stroke rate of 2.6% [41]. Another strictly endovascular technique to extend the landing zone more proximally is the use of branched or fenestrated stent grafts. Triple-branched stent grafts have been demonstrated to be viable in the case of acute non-A non-B aortic dissection with satisfactory results [42,43]. In a case report by Spath et al., physician-modified fenestrated stent graft was also shown to yield promising results even in the case of undetected, complex multiorgan malperfusion [44].

### 4.3. Notable Mention: Other Hybrid Repair

While not mentioned in current guidelines in the context of non-A non-B dissections, hybrid repair utilizing zone 0 TEVAR after first rerouting the branches of the aortic arch has been used extensively in some centers, while completely disregarded in others as demonstrated by Carino et al. [34]. However, these hybrid methods have been shown to carry a high risk of retrograde type A dissection, increased complexity of treatment, and increased hazard of stroke and mortality [34,45,46].

Another novel surgical technique is the so-called aortic arch inclusion technique, where an FET prosthesis is trimmed to a shorter length, leaving an opening for the supra-aortic vessels. The prosthesis is then inserted into the aortic arch through a small incision with the endovascular stent graft portion of the prosthesis fulfilling its original purpose, while the stent-free section is sutured to the inside of the aortic arch. As the graft was trimmed beforehand, supra-aortic vessels remain uncovered and untouched throughout the operation [47]. Early results of a limited patient cohort with this technique seem promising, especially in the setting of non-A non-B dissection. However, more data still need to be obtained before reaching a verdict on this rare technique. Additionally, as this technique does not replace the whole aortic arch, the primary entry tear needs to be reachable by the trimmed dacron graft used in the technique, further limiting its indication [48,49].

## 5. Conclusions

In conclusion, the management of non-A non-B dissection continues to be a subject of considerable debate, primarily due to the reliance on retrospective data, which limits the strength of the evidence base. As an emerging disease pattern, primarily recognized through the TEM classification, non-A non-B dissection poses unique challenges that necessitate ongoing research and clinical exploration. Despite the controversies surrounding its treatment, current consensus guidelines suggest that the frozen elephant trunk procedure is the preferred approach in most surgical cases. As further studies are conducted, a clearer understanding of this condition may emerge, potentially refining treatment strategies and enhancing patient outcomes.

## Abbreviations

TEM	type of dissection, entry site, and malperfusion
EACTS/STS	European association of cardiothoracic surgery/Society of thoracic surgeons
ESC	European society of cardiology
CTA	Computed tomography angiography
FET	Frozen elephant trunk
SACP	selective antegrade cerebral perfusion
dSINE	distal stent graft induced new entry
TEVAR	thoracic endovascular aortic repair
PTFE	polytetrafluoroethylene

## References

[1] Pasic M., Knollman F., Hetzer R. (1999). Isolated non-A, non-B dissection of the aortic arch. N. Engl. J. Med..

[2] Czerny M., Grabenwoger M., Berger T., Aboyans V., Della Corte A., Chen E.P., Desai N.D., Dumfarth J., Elefteriades J.A., Etz C.D. (2024). EACTS/STS Guidelines for diagnosing and treating acute and chronic syndromes of the aortic organ. Eur. J. Cardiothorac. Surg..

[3] Mazzolai L., Teixido-Tura G., Lanzi S., Boc V., Bossone E., Brodmann M., Bura-Riviere A., De Backer J., Deglise S., Della Corte A. (2024). 2024 ESC Guidelines for the management of peripheral arterial and aortic diseases. Eur. Heart J..

[4] Sievers H.H., Rylski B., Czerny M., Baier A.L.M., Kreibich M., Siepe M., Beyersdorf F. (2020). Aortic dissection reconsidered: Type, entry site, malperfusion classification adding clarity and enabling outcome prediction. Interact. Cardiovasc. Thorac. Surg..

[5] Rylski B., Perez M., Beyersdorf F., Reser D., Kari F.A., Siepe M., Czerny M. (2017). Acute non-A non-B aortic dissection: Incidence, treatment and outcome. Eur. J. Cardiothorac. Surg..

[6] Trimarchi S., de Beaufort H.W.L., Tolenaar J.L., Bavaria J.E., Desai N.D., Di Eusanio M., Di Bartolomeo R., Peterson M.D., Ehrlich M., Evangelista A. (2019). Acute aortic dissections with entry tear in the arch: A report from the International Registry of Acute Aortic Dissection. J. Thorac. Cardiovasc. Surg..

[7] Urbanski P.P., Wagner M. (2016). Acute non-A-non-B aortic dissection: Surgical or conservative approach?. Eur. J. Cardiothorac. Surg..

[8] Berger T., Maier A., Kletzer J., Schlett C.L., Kondov S., Czerny M., Rylski B., Kreibich M. (2024). Radiographic complicated and uncomplicated descending aortic dissections: Aortic morphological differences by CT angiography and risk factor analysis. Eur. Heart J. Cardiovasc. Imaging.

[9] Wada T., Yamamoto H., Takagi D., Kadohama T., Yamaura G., Kiryu K., Igarashi I. (2022). Aortic remodeling, reintervention, and survival after zone 0 arch repair with frozen elephant trunks for acute type A aortic dissection: Midterm results. JTCVS Tech..

[10] Inoue Y., Minatoya K., Oda T., Itonaga T., Seike Y., Tanaka H., Sasaki H., Kobayashi J. (2016). Surgical outcomes for acute type A aortic dissection with aggressive primary entry resection. Eur. J. Cardiothorac. Surg..

[11] Berger T., Chikvatia S., Siepe M., Kondov S., Meissl D., Gottardi R., Rylski B., Czerny M., Kreibich M. (2023). Concomitant aortic root replacement during frozen elephant trunk implantation does not increase perioperative risk. Eur. J. Cardiothorac. Surg..

[12] Berger T., Kreibich M., Rylski B., Schibilsky D., Pooth J.S., Fagu A., Zimmer E., Pingpoh C., Beyersdorf F., Czerny M. (2022). Composition of the surgical team in aortic arch surgery-a risk factor analysis. Eur. J. Cardiothorac. Surg..

[13] Berger T., Kreibich M., Rylski B., Morlock J., Kondov S., Scheumann J., Kari F.A., Staier K., Maier S., Beyersdorf F. (2020). Evaluation of myocardial injury, the need for vasopressors and inotropic support in beating-heart aortic arch surgery. J. Cardiovasc. Surg..

[14] Papantchev V., Stoinova V., Aleksandrov A., Todorova-Papantcheva D., Hristov S., Petkov D., Nachev G., Ovtscharoff W. (2013). The role of Willis circle variations during unilateral selective cerebral perfusion: A study of 500 circles. Eur. J. Cardiothorac. Surg..

[15] Kreibich M., Siepe M., Berger T., Kondov S., Morlock J., Pingpoh C., Beyersdorf F., Rylski B., Czerny M. (2021). The Frozen Elephant Trunk Technique for the Treatment of Type B and Type Non-A Non-B Aortic Dissection. Eur. J. Vasc. Endovasc. Surg..

[16] Wisniewski K., Dell’Aquila A.M., Motekallemi A., Oberhuber A., Schafers J.F., Marchiori E., Weber R., Martens S., Rukosujew A. (2024). The frozen elephant trunk technique in acute aortic dissection: The ultimate solution? An institutional experience. Front. Cardiovasc. Med..

[17] Berger T., Kreibich M., Mueller F., Breurer-Kellner L., Rylski B., Kondov S., Schrofel H., Pingpoh C., Beyersdorf F., Siepe M. (2022). Risk factors for stroke after total aortic arch replacement using the frozen elephant trunk technique. Interact. Cardiovasc. Thorac. Surg..

[18] Wisniewski K., Motekallemi A., Dell’Aquila A.M., Oberhuber A., Schaefers J.F., Ibrahim A., Martens S., Rukosujew A. (2022). Single-Center Experience With the Thoraflex Hybrid Prosthesis: Indications, Implantation Technique and Results. Front. Cardiovasc. Med..

[19] Rylski B., Blanke P., Beyersdorf F., Desai N.D., Milewski R.K., Siepe M., Kari F.A., Czerny M., Carrel T., Schlensak C. (2014). How does the ascending aorta geometry change when it dissects?. J. Am. Coll. Cardiol..

[20] Kreibich M., Berger T., Morlock J., Kondov S., Scheumann J., Kari F.A., Rylski B., Siepe M., Beyersdorf F., Czerny M. (2018). The frozen elephant trunk technique for the treatment of acute complicated Type B aortic dissection. Eur. J. Cardiothorac. Surg..

[21] Czerny M., Eggebrecht H., Rousseau H., Mouroz P.R., Janosi R.A., Lescan M., Schlensak C., Bockler D., Ante M., Weijde E.V. (2020). Distal Stent Graft-Induced New Entry After TEVAR or FET: Insights Into a New Disease From EuREC. Ann. Thorac. Surg..

[22] Luo C., Qi R., Zhong Y., Chen S., Liu H., Guo R., Ge Y., Sun L., Zhu J. (2021). Early and Long-Term Follow-Up for Chronic Type B and Type Non-A Non-B Aortic Dissection Using the Frozen Elephant Trunk Technique. Front. Cardiovasc. Med..

[23] Liu J., Yang F., Chen L., Xie E., Su S., Liu Y., Geng Q., Fan R., Li J., Luo J. (2022). Management and Outcomes of Non-A Non-B Aortic Dissection. Eur. J. Vasc. Endovasc. Surg..

[24] Berger T., Kreibich M., Morlock J., Kondov S., Scheumann J., Kari F.A., Rylski B., Siepe M., Beyersdorf F., Czerny M. (2018). True-lumen and false-lumen diameter changes in the downstream aorta after frozen elephant trunk implantation. Eur. J. Cardiothorac. Surg..

[25] Kreibich M., Berger T., Rylski B., Chen Z., Beyersdorf F., Siepe M., Czerny M. (2020). Aortic reinterventions after the frozen elephant trunk procedure. J. Thorac. Cardiovasc. Surg..

[26] Kreibich M., Bunte D., Berger T., Votsch A., Rylski B., Krombholz-Reindl P., Chen Z., Morlock J., Beyersdorf F., Winkler A. (2020). Distal Stent Graft-Induced New Entries After the Frozen Elephant Trunk Procedure. Ann. Thorac. Surg..

[27] Berger T., Graap M., Rylski B., Fagu A., Gottardi R., Walter T., Discher P., Hagar M.T., Kondov S., Czerny M. (2022). Distal Aortic Failure Following the Frozen Elephant Trunk Procedure for Aortic Dissection. Front. Cardiovasc. Med..

[28] Berger T., Kreibich M., Rylski B., Kondov S., Fagu A., Beyersdorf F., Siepe M., Czerny M. (2021). The 3-step approach for the treatment of multisegmental thoraco-abdominal aortic pathologies. Interact. Cardiovasc. Thorac. Surg..

[29] Kreibich M., Siepe M., Berger T., Kondov S., Morlock J., Pingpoh C., Beyersdorf F., Rylski B., Czerny M. (2022). Downstream thoracic endovascular aortic repair following zone 2, 100-mm stent graft frozen elephant trunk implantation. Interact. Cardiovasc. Thorac. Surg..

[30] Walter T., Berger T., Kondov S., Gottardi R., Benk J., Rylski B., Czerny M., Kreibich M. (2022). Postoperative In-Stent Thrombus Formation Following Frozen Elephant Trunk Total Arch Repair. Front. Cardiovasc. Med..

[31] El-Sayed Ahmad A., Silaschi M., Borger M., Seidiramool V., Hamiko M., Leontyev S., Zierer A., Doss M., Etz C.D., Benedikt P. (2023). The Frozen Elephant Technique Using a Novel Hybrid Prosthesis for Extensive Aortic Arch Disease: A Multicentre Study. Adv. Ther..

[32] Folkmann S., Arnold Z., Geisler D., Lenz V., Miosga D., Harrer M., Trnka H., Eller R., Aschacher T., Winkler B. (2024). First-in-men experience with a novel frozen elephant trunk prosthesis featuring an endovascular side branch for left subclavian artery connection. Eur. J. Cardiothorac. Surg..

[33] Borst H.G., Walterbusch G., Schaps D. (1983). Extensive aortic replacement using "elephant trunk" prosthesis. Thorac. Cardiovasc. Surg..

[34] Carino D., Singh M., Molardi A., Agostinelli A., Goldoni M., Pacini D., Nicolini F. (2019). Non-A non-B aortic dissection: A systematic review and meta-analysis. Eur. J. Cardiothorac. Surg..

[35] Kosiorowska M., Berezowski M., Widenka K., Kreibich M., Beyersdorf F., Czerny M., Rylski B. (2022). Non-A non-B acute aortic dissection with entry tear in the aortic arch. Interact. Cardiovasc. Thorac. Surg..

[36] Nucera M., Kreibich M., Yildiz M., Berger T., Kolb R.K., Kondov S., Kunzmann S., Rylski B., Makaloski V., Siepe M. (2024). Endovascular aortic repair in patients with Marfan and Loeys-Dietz syndrome is safe and durable when employed by a multi-disciplinary aortic team. Eur. J. Cardiothorac. Surg..

[37] Le Huu A., Olive J.K., Cekmecelioglu D., Chatterjee S., Amarasekara H.S., Green S.Y., Coselli J.S., Preventza O. (2022). Endovascular therapy for patients with heritable thoracic aortic disease. Ann. Cardiothorac. Surg..

[38] Morasch M.D. (2009). Technique for subclavian to carotid transposition, tips, and tricks. J. Vasc. Surg..

[39] Voigt S.L., Bishawi M., Ranney D., Yerokun B., McCann R.L., Hughes G.C. (2019). Outcomes of carotid-subclavian bypass performed in the setting of thoracic endovascular aortic repair. J. Vasc. Surg..

[40] Ahmad W., Mylonas S., Majd P., Brunkwall J.S. (2017). A current systematic evaluation and meta-analysis of chimney graft technology in aortic arch diseases. J. Vasc. Surg..

[41] Hogendoorn W., Schlosser F.J., Moll F.L., Sumpio B.E., Muhs B.E. (2013). Thoracic endovascular aortic repair with the chimney graft technique. J. Vasc. Surg..

[42] Burysz M., Milnerowicz A., Bartus K., Litwinowicz R. (2023). Total endovascular repair of an aortic arch using a triple-branched graft in acute non-A non-B aortic dissection. Kardiol. Pol..

[43] Li Q., Wu Q., Wu W., Dai X., Fang G., Xie X., Chen L. (2023). Triple-Branched Stent Graft Implantation for Acute Non-A-non-B Aortic Dissection. Ann. Thorac. Surg..

[44] Spath P., Stana J., Marazzi G., Peterss S., Fernandez-Prendes C., Tsilimparis N. (2023). Emergent physician modified carotid fenestrated TEVAR for the treatment of a complicated acute type nonA-nonB aortic dissection with undetected multiorgan malperfusion. J. Cardiovasc. Surg..

[45] Czerny M., Weigang E., Sodeck G., Schmidli J., Antona C., Gelpi G., Friess T., Klocker J., Szeto W.Y., Moeller P. (2012). Targeting landing zone 0 by total arch rerouting and TEVAR: Midterm results of a transcontinental registry. Ann. Thorac. Surg..

[46] Wallen T., Carter T., Habertheuer A., Badhwar V., Jacobs J.P., Yerokun B., Wallace A., Milewski K., Szeto W.Y., Bavaria J.E. (2021). National Outcomes of Elective Hybrid Arch Debranching with Endograft Exclusion versus Total Arch Replacement Procedures: Analysis of the Society of Thoracic Surgeons Adult Cardiac Surgery Database. Aorta.

[47] Liu K., Zhu C., Zheng X., Wang T., Xu R., Zhu Z., Li D., Piao H., Li B., Wang Y. (2020). A New Aortic Arch Inclusion Technique With Frozen Elephant Trunk for Type A Aortic Dissection. Ann. Surg..

[48] Li Q., Li B., Xi S., Li Z., Zhu Z., Jin Z., Yang F., Liu L. (2024). Experience with aortic arch inclusion technique using artificial blood vessel for type A aortic dissection: An application study. J. Cardiothorac. Surg..

[49] Wang W., Piao H., Wang Y., Li B., Zhu Z., Wang T., Liu K. (2022). Early outcomes with a hybrid technique for repair of a non-A non-B aortic dissection. J. Thorac. Cardiovasc. Surg..

